# Dissociable endogenous and exogenous attention in disorders of consciousness^☆^

**DOI:** 10.1016/j.nicl.2013.10.008

**Published:** 2013-10-16

**Authors:** Srivas Chennu, Paola Finoia, Evelyn Kamau, Martin M. Monti, Judith Allanson, John D. Pickard, Adrian M. Owen, Tristan A. Bekinschtein

**Affiliations:** a Division of Neurosurgery, University of Cambridge, Box 167, Level 4, A Block, Addenbrooke's Hospital, Hills Road, Cambridge CB2 0QQ, UK; b Department of Psychology, University of California at Los Angeles, 1285 Franz Hall, Box 951563, Los Angeles, CA 90095-1563, USA; c Cambridge University Hospitals NHS Foundation Trust, Box 120, Addenbrooke's Hospital, Hills Road, Cambridge CB2 0QQ, UK; d The Brain and Mind Institute, Room 225, Natural Sciences Centre, The University of Western Ontario, London, Ontario N6A 5B7, Canada; e Medical Research Council, Cognition and Brain Sciences Unit, 15 Chaucer Road, Cambridge CB2 7EF, UK

**Keywords:** Vegetative state, Minimally conscious state, Attention, Consciousness, Electroencephalography

## Abstract

Recent research suggests that despite the seeming inability of patients in vegetative and minimally conscious states to generate consistent behaviour, some might possess covert awareness detectable with functional neuroimaging. These findings motivate further research into the cognitive mechanisms that might support the existence of consciousness in these states of profound neurological dysfunction. One of the key questions in this regard relates to the nature and capabilities of attention in patients, known to be related to but distinct from consciousness. Previous assays of the electroencephalographic P300 marker of attention have demonstrated its presence and potential clinical value. Here we analysed data from 21 patients and 8 healthy volunteers collected during an experimental task designed to engender exogenous or endogenous attention, indexed by the P3a and P3b components, respectively, in response to a pair of word stimuli presented amongst distractors. Remarkably, we found that the early, bottom-up P3a and the late, top-down P3b could in fact be dissociated in a patient who fitted the behavioural criteria for the vegetative state. In juxtaposition with healthy volunteers, the patient's responses suggested the presence of a relatively high level of attentional abilities despite the absence of any behavioural indications thereof. Furthermore, we found independent evidence of covert command following in the patient, as measured by functional neuroimaging during tennis imagery. Three other minimally conscious patients evidenced non-discriminatory bottom-up orienting, but no top-down engagement of selective attentional control. Our findings present a persuasive case for dissociable attentional processing in behaviourally unresponsive patients, adding to our understanding of the possible levels and applications of consequent conscious awareness.

## Highlights

• We dissociated endogenous and exogenous attention in disorders of consciousness.
• 21 vegetative (VS) and minimally conscious (MCS) patients tested with EEG task
• Exogenous bottom-up P3a found in 3 minimally conscious and 1 vegetative patient
• Endogenous top-down P3b found in one VS patient who performed fMRI tennis imagery
• Discriminative attention can be preserved in disorders of consciousness.

## 1 Introduction

The last decade has seen significant advances in the application of modern neuroimaging and electrophysiology for improving our understanding of chronic Disorders of Consciousness (DoC). These neurological disorders, often brought on by severe traumatic brain injury or hypoxia, encompass the Vegetative State (VS), and the Minimally Conscious State (MCS) (Giacino et al., 2002; Jennett and Plum, 1972; Laureys et al., 2010), and are characterised by varying degrees of ‘wakefulness without awareness’ (Cruse et al., 2011a). The significant clinical uncertainties surrounding diagnosis and prognosis for these patients continue to present a societal challenge with serious ethical implications.

However, despite the seeming inability of patients to generate consistent behaviour, a considerable amount of recent evidence suggests that some patients in these states might possess covert awareness detectable with fMRI (functional Magnetic Resonance Imaging) and cognitive EEG (Electroencephalography) (Bardin et al., 2011; Bekinschtein et al., 2009a; Cruse et al., 2011b, 2012a, 2012b; Faugeras et al., 2011, 2012; Goldfine et al., 2011; Monti et al., 2009, 2010; Owen et al., 2006; Schnakers et al., 2008). These findings are certainly encouraging, and motivate more detailed research into the cognitive mechanisms that might support the existence of consciousness in patients with prolonged disorders of consciousness.

One of the key questions in this regard relates to the nature and capabilities of attention in patients, a very well studied cognitive process we know to be related to, but distinct from, consciousness (Boxtel et al., 2010; Koch and Tsuchiya, 2007). Studies of high-level cognitive function like command following in DoC implicitly rely on the patient's ability to pay attention to and follow task instructions (Cruse et al., 2011b, 2012b; Goldfine et al., 2011; Monti et al., 2010; Owen et al., 2006). However, the direct measurement of attention in patients is valuable in its own right, and could even be used for efficient communication (Naci et al., 2013). Early EEG studies with DoC patients attempted to specifically detect the presence of attentional capabilities by measuring the P300 Event-Related Potential (ERP), well-understood as a correlate of attention and conscious perception (Rappaport et al., 1991; Reuter et al., 1989; Witzke and Schönle, 1996). Building upon this work, more recent research has investigated patients' abilities to generate P300 responses to oddball and own-name stimuli (Fischer et al., 2010; Kotchoubey et al., 2001; Lulé et al., 2012; Perrin et al., 2006; Schnakers et al., 2008).

Alongside, extensive research on attention involving healthy populations has deconstructed the P300 response into separable subcomponents represented by the P3a and P3b. The relatively earlier, frontally centred *novelty* P3a is thought to index *exogenous* attention, triggered by ‘bottom-up’ stimulus novelty that may be task-irrelevant. The later, parietally focused *target* P3b, on the other hand, is seen as a marker of ‘top-down’ or volitional engagement of *endogenous* attention to task-relevant targets to be consolidated into working memory and made available for conscious access (Comerchero and Polich, 1999; Courchesne et al., 1975; Friedman et al., 2001; Katayama and Polich, 1998; Polich, 1988, 2007; Polich and Criado, 2006; Squires et al., 1975). These subcomponents are also thought to have distinct cortical generators, with the P3a having an anterior contribution, while the P3b is thought to be more distributed, generated by frontal, parietal and temporo-occipital regions (Friedman et al., 2001; Polich, 2007; Volpe et al., 2007). In addition, we also know that while the P3a can still be observed during natural non-REM sleep (Cote, 2002) and sedation (Koelsch et al., 2006), the P3b is severely attenuated in these states, suggesting that while involuntary orienting to exogenous stimuli is preserved, volitional attentional engagement is absent (see Chennu and Bekinschtein, 2012 for an integrative review). Taken together, these findings lend support to the bottom-up vs. top-down distinction between these subcomponents of the P300.

In this article, we draw upon these insights provided by the literature on the distinction between these processes (see Friedman et al. (2001) and Polich (2007) for reviews) to investigate them in DoC. Previous assays of the P300 ERP in patients have demonstrated its presence and potential clinical value, but here we go further, and show that the P3a and P3b can in fact be dissociated in DoC patients. We recorded high-density EEG in conjunction with an experimental task that engendered either exogenous or endogenous attention in response to a pair of word stimuli. Specifically, depending on task instructions that varied by block, one of a pair of equiprobable words presented amongst distractors was designated as the *explicit* target, while the other became a salient *implicit* target. In healthy adults, we found that while explicit targets elicited an endogenous P3b, implicit targets elicited an exogenous P3a. Remarkably, a similar pattern of responses was evoked in a patient who fit the behavioural criteria for the vegetative state, suggesting that dissociable mechanisms of bottom-up and top-down attention can potentially be preserved after severe brain injury. Interestingly, this patient had relatively preserved cortical structural integrity and was also able to generate independent evidence of covert volitional abilities measured by fMRI, when asked to follow commands during the tennis imagery task (Monti et al., 2010; Owen et al., 2006). Our cohort analysis also identified MCS patients who generated ERPs indicative of exogenous attentional orienting, but did not show evidence of top-down endogenous attention. These findings provide new insights into residual attentional capabilities of patients, and complement promising neuroimaging research into the presence of covert conscious awareness in DoC.

## 2 Methods

### 2.1 Participants

#### 2.1.1 Healthy volunteers

8 neurologically healthy adults (3 male; 5 female) with normal binaural hearing (mean age = 27.9; s.d. = 4.1) participated in the study. They gave written informed consent and were paid 10 GBP per hour for their time. Ethical approval for testing healthy volunteers was provided by the Cambridge Psychology Research Ethics Committee.

#### 2.1.2 Patients

A convenience sample of 30 VS or MCS patients, assessed at Addenbrooke's Hospital in Cambridge (UK) between September 2011 and June 2013 were included in the study. Written informed consent was acquired from all patients' families and medical teams. Ethical approval for testing patients was provided by the National Research Ethics Service (National Health Service, UK).

EEG data acquired from 9 patients were rejected due to excessive noise artefact. Demographic details of the remaining 21 patients, data from whom was analysed, are listed in Table 1.

Patients were admitted for 4–5 days as part of a comprehensive testing protocol that included the EEG task described below, in addition to the fMRI tennis imagery task described by Owen et al. (2006). Patients were assessed with the Coma Recovery Scale—Revised (CRS-R) (Kalmar and Giacino, 2005) everyday during their admission. As listed in Table 1, the highest CRS-R score observed across all assessments of each patient was used to assign a diagnosis of VS or MCS. Of the 21 patients, 9 were diagnosed to be VS, with CRS-R scores between 7 and 8. The 12 other patients diagnosed as MCS had a wide range of scores between 8 and 19.

We also assessed the degree of integrity of cortical structure by detailed visual evaluation of T1-weighted anatomical MRI images of patients, using cortical atrophy scoring criteria previously adapted for this patient group (Bekinschtein et al., 2008, 2009b). No pre-processing (segmentation, normalisation, classification, etc.) of the images was performed prior to the scoring, as these steps can, in many instances, distort abnormal structural MRI scans of DoC patients, with unexpected outcomes. This rating scale assigned a score of 0 (no atrophy), 1 (very low), 2 (mild), 3 (severe) and 4 (highly severe atrophy) to each patient, as listed in Table 1. The rating was conducted blind to all other clinical and neuroimaging measures listed therein.

### 2.2 Stimuli

**Fig. S1:**
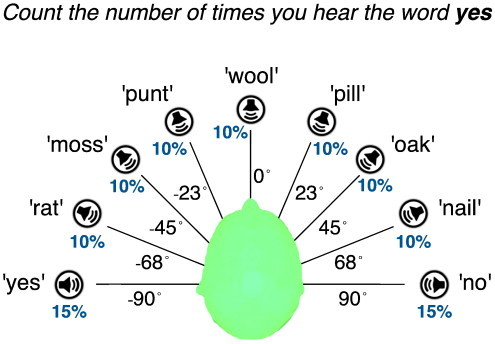
Apparent auditory lateralisation and frequency of experimental stimuli. The words ‘YES’ and ‘NO’ were presented at apparent spatial orientations of − 90° and ± 90° respectively, and each occurred approximately 15% of the time within an experimental block. One of the two words was randomly designated as the explicit target for the block, while the other served as the implicit target. Randomly selected distractor words from the list in Inline Supplementary Table S1 were presented equally randomly at one of seven spatial orientations from − 68° to + 68°, with each orientation being selected approximately 10% of time in a block.

The experiment comprised of 20 blocks (lasting approx. 1.5 min each) of binaurally presented word stimuli digitised at 44 kHz, played at a hearing volume of approximately 85 dB SPL. A block consisted of 90–100, emotionally neutral, monosyllabic words spoken by a female native English speaker, presented once every 900–1100 ms. Of these, approx. 66−71 words were irrelevant distractors, selected from a pre-specified list of 50 words listed in Inline Supplementary Table S1, previously employed in an fMRI study with DoC patients (Monti et al., 2009). These distractors were presented in a randomly permuted order, ensuring that the same word was not presented in quick succession. On average, a given distractor word was presented 1.3–1.4 times in a block. The apparent spatial orientation of the auditory source of the distractor words was manipulated by introducing an interaural timing difference (ITD) between the onset of the left and right audio channels. This ITD was randomly selected amongst − 495 μs, − 330 μs, − 165 μs, 0 μs, 165 μs, 330 μs or 495 μs for each distractor word (Feddersen et al., 1957; Moore, 2003), to produce a linear mapping onto apparent orientations of − 68°, − 45°, − 23°, 0°, 23°, 45°or 68° in auditory space (see Inline Supplementary Fig. S1 for a visual illustration).

The experiment comprised of 20 blocks (lasting approx. 1.5 min each) of binaurally presented word stimuli digitised at 44 kHz, played at a hearing volume of approximately 85 dB SPL. A block consisted of 90–100, emotionally neutral, monosyllabic words spoken by a female native English speaker, presented once every 900–1100 ms. Of these, approx. 66 − 71 words were irrelevant distractors, selected from a pre-specified list of 50 words listed in Inline Supplementary Table S1, previously employed in an fMRI study with DoC patients (Monti et al., 2009). These distractors were presented in a randomly permuted order, ensuring that the same word was not presented in quick succession. On average, a given distractor word was presented 1.3–1.4 times in a block. The apparent spatial orientation of the auditory source of the distractor words was manipulated by introducing an interaural timing difference (ITD) between the onset of the left and right audio channels. This ITD was randomly selected amongst − 495 μs, − 330 μs, − 165 μs, 0 μs, 165 μs, 330 μs or 495 μs for each distractor word (Feddersen et al., 1957; Moore, 2003), to produce a linear mapping onto apparent orientations of − 68°, − 45°, − 23°, 0°, 23°, 45°or 68° in auditory space (see Inline Supplementary Fig. S1 for a visual illustration).

Inline Supplementary Figure S1

Inline Supplementary Fig. S1 can be found online at http://dx.doi.org/10.1016/j.nicl.2013.10.008.

Inline Supplementary Table S1

**Table S1:** *Target* and distractor words used in the experimental task. Target words are indicated in italics.

Word	Duration (ms)	Word	Duration (ms)
*Yes*	525	Rat	618
*No*	520	Rice	637
Gown	571	Robe	543
Mop	576	Rock	587
Moss	590	Roof	591
Moth	499	Rope	501
Mug	542	Rug	540
Nail	595	Shed	554
Newt	637	Ship	525
Oak	470	Sock	633
Oat	537	Tape	520
Owl	599	Toad	543
Ox	572	Tram	579
Pan	569	Tub	455
Pear	503	Van	596
Pea	480	Vat	592
Pen	450	Vine	638
Pig	490	Wasp	627
Pill	528	Wig	549
Pine	607	Wine	535
Pin	478	Wolf	521
Plum	537	Wood	443
Pram	552	Wool	572
Punt	582	Worm	625
Raft	639	Wren	507
Rake	600	Yak	540
		Mean	554

Inline Supplementary Table S1 Target and distractor words used in the experimental task.

Inline Supplementary Table S1 can be found online at http://dx.doi.org/10.1016/j.nicl.2013.10.008.

Randomly interspersed amongst the distractors were the words ‘YES’ and ‘NO’, each presented between 13 and 16 times during a block. Consecutive presentations of these words were separated by 2–8 distractors. The word YES was always presented at an apparent spatial orientation of − 90° (ITD = -660 μs, i.e., to the left ear), and the word NO at + 90° (ITD = 660 μs, i.e., to the right ear). This ensured that targets had spatial orientations that were discernably distinct from distractors, allowing participants to beneficially allocate auditory spatial attention to the targets. All healthy volunteers reported perceiving the differing auditory lateralisations of the targets and distractors.

At the beginning of each block, either YES or NO was randomly designated by auditory instruction as the *explicit target* word for that block. Subjects were asked to count occurrences of this word, and hence it became *endogenously* task-relevant, due to the top-down expectation of its occurrence. If the word YES was designated as the explicit target for a block, then the word NO became the *implicit target* for that block, and vice versa. This implicit target was *exogenously* salient due to its relatively higher frequency than distractor words. Over a full experimental run, half the blocks had YES as the explicit target and the other half had NO, and the order of the blocks was randomised.

### 2.3 Experimental task

The auditory word stimuli were presented with Etymotics ER-3A in-ear phones at a comfortable volume level. Each block began with a 6-second pause, followed by a short beep and then the auditory instruction “*Count the number of times you hear the word* [*YES*/*NO*]”. Approximately 3 s after the end of the instruction, the presentation of words began as described above. At the end of each block, there was a long beep to indicate the end of the word stimuli. Before beginning the next block, healthy volunteers were asked to indicate the number of explicit target words they had counted with a button press. In case of patients, the next block began automatically, after a 10-second break. Both healthy volunteers and patients were asked to stay awake and alert during the testing sessions and perform the experimental task requested. The behaviourally apparent arousal levels of patients were monitored during the session to try and ensure that they stayed awake, but this could not be reliably assessed in all cases.

### 2.4 EEG data collection and analysis

**Fig. 1:**
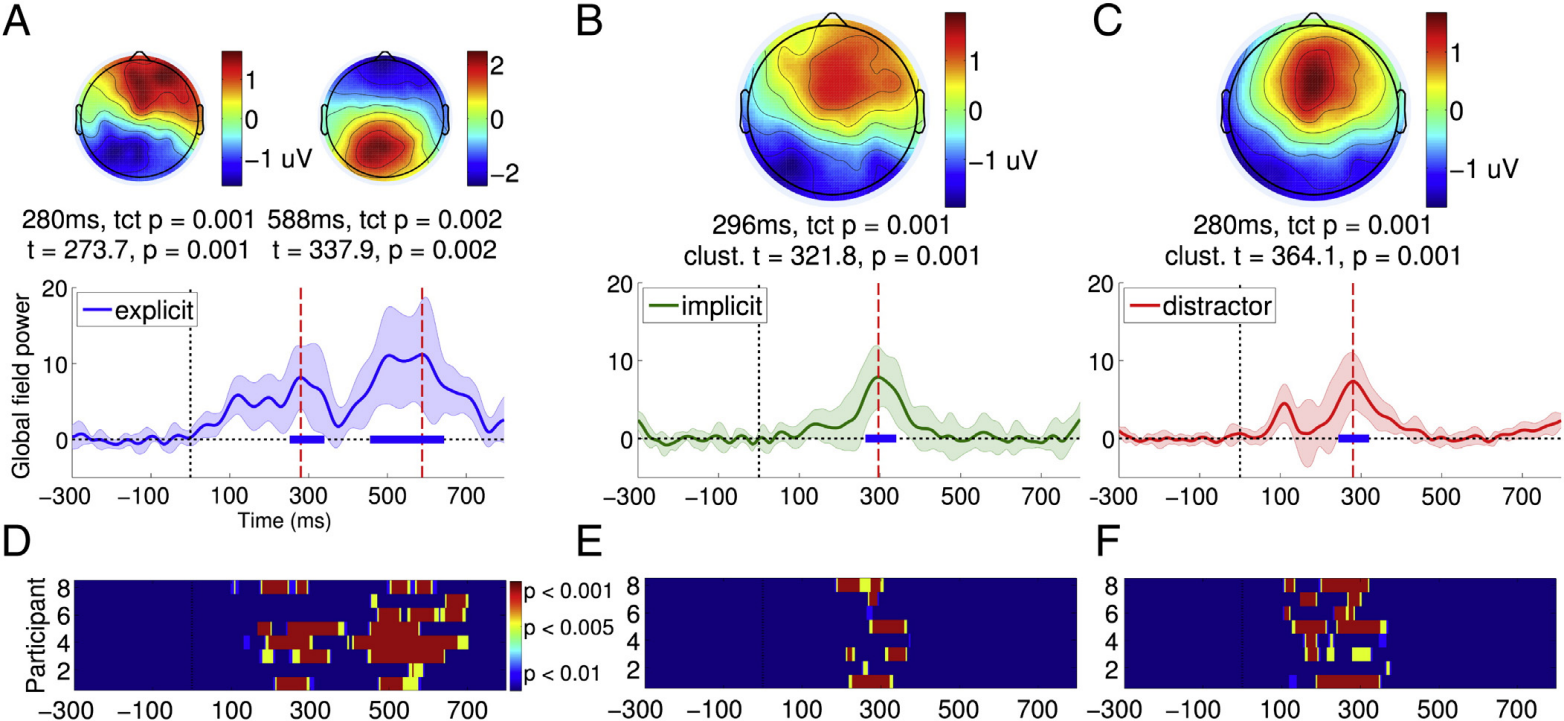
Responses to targets and distractors in healthy volunteers. In panels A–C, bottom half plots time course of GFP of an ERP grand average across healthy volunteers. The shaded region alongside indicates the standard deviation of these GFPs across participants. The horizontal thick blue line indicates the temporal extent of a statistically significant cluster (across participants) of contiguous time points where GFP was greater than baseline. The vertical red dashed line indicates the time point within the cluster at which GFP was maximal, and the upper half of the panel plots the scalp topography of the ERP at this time point. The time point itself is mentioned in the text below, along with the mean t-value and p-value of the cluster. Explicit targets (panel A) elicited a frontal P3a within the 100–400 ms followed by a parietal P3b within the 400–700 ms. Implicit targets and distractors (panels B and C) only evoked a frontal P3a within 100–400 ms. Panels D–F plot stacked colour maps of statistically significant clusters of GFP observed in individual participants. Each horizontal line plots the significant time course of an individual participant's GFP on a colour scale. Panel D plots GFP clusters within the 100–400 ms P3a window and the 400–700 ms P3b window for explicit targets. Similarly, panels E and F plot clusters within the 100–400 ms window for implicit targets and distractors, respectively.

During the experiment, 128-channel high-density EEG data in microvolts (μV), sampled at 250 Hz and referenced to the vertex, were collected using the Net Amps 300 amplifier (Electrical Geodesics Inc., Oregon, USA). Data from 91 channels over the scalp surface (at locations shown in Fig. 1B, top) were retained for further analysis. Channels on the neck, cheeks and forehead, which mostly contributed more movement-related noise than signal in patients, were excluded. The retained continuous data were low-pass filtered at 20 Hz, high-pass filtered at 0.5 Hz, and epoched between − 300 and 800 ms relative to the start of the presentation of each word. The epochs generated were baseline-corrected relative to the mean activity during the − 300−0 ms window.

Data containing excessive eye movement or muscular artefact were rejected by a quasi-automated procedure: noisy channels and epochs were identified by calculating their normalised variance and then manually rejected or retained by visual confirmation. Independent Components Analysis (ICA) based on the Infomax ICA algorithm (Bell and Sejnowski, 1995) was used to visually identify and reject noisy components. Finally, previously rejected channels were interpolated using spherical spline interpolation, and data were re-referenced to the average of all channels. These processing steps were implemented using custom MATLAB scripts based on EEGLAB (Delorme and Makeig, 2004). The number of channels interpolated, epochs and ICA components rejected in healthy volunteer and patient datasets discussed in the Results and discussion section are listed in Inline Supplementary Table S2. Also specified therein are the numbers of explicit target, implicit target and distractor trials available for the statistical analysis procedure described next.

Data containing excessive eye movement or muscular artefact were rejected by a quasi-automated procedure: noisy channels and epochs were identified by calculating their normalised variance and then manually rejected or retained by visual confirmation. Independent Components Analysis (ICA) based on the Infomax ICA algorithm (Bell and Sejnowski, 1995) was used to visually identify and reject noisy components. Finally, previously rejected channels were interpolated using spherical spline interpolation, and data were re-referenced to the average of all channels. These processing steps were implemented using custom MATLAB scripts based on EEGLAB (Delorme and Makeig, 2004). The number of channels interpolated, epochs and ICA components rejected in healthy volunteer and patient datasets discussed in the Results and discussion section are listed in Inline Supplementary Table S2. Also specified therein are the numbers of explicit target, implicit target and distractor trials available for the statistical analysis procedure described next.

Inline Supplementary Table S2 **Results of data preprocessing in healthy volunteers (HV1-8) and selected patients (P1, P10, P11 and P20).**

**Table S2:** Results of data preprocessing in healthy volunteers (HV1-8) and selected patients (P1, P10, P11 and P20).

Subject	Channels rejected	Trials rejected	ICA components rejected	Explicit target trials	Implicit target trials	Distractor trials
HV1	0	12	26	234	233	441
HV2	17	20	22	237	232	633
HV3	9	4	21	241	241	632
HV4	3	0	23	237	237	669
HV5	2	0	20	243	243	660
HV6	2	0	10	291	291	487
HV7	1	6	9	290	291	489
HV8	5	9	7	293	292	542
P1	0	9	5	288	288	489
P10	11	11	41	294	295	474
P11	0	191	28	282	282	479
P20	2	58	47	277	280	514

Inline Supplementary Table S2

Inline Supplementary Table S2 can be found online at http://dx.doi.org/10.1016/j.nicl.2013.10.008.

Epochs from an experimental condition and its own baseline period, or pairs of conditions of interest, were compared using a non-parametric t-test based on that employed in the FieldTrip toolbox (Maris and Oostenveld, 2007). This test identified temporal clusters of statistically significant differences between the Global Field Power (GFP) (Lehmann and Skrandies, 1980; Murray et al., 2008; Skrandies, 1990) of the ERPs in the two conditions using a Monte Carlo procedure for estimating p-values. To elaborate, we first calculated ERPs by separately averaging epochs (for single-subject analysis) or subject-wise averages (for group analysis) included in each condition. The difference between the GFP time courses of the two ERPs was then tested for statistical significance using a randomisation testing procedure. To do this, the original epochs/subject-wise averages were mixed together and separated into two new sets that contained random samples from the original conditions. These sets were again separately averaged to calculate new ERPs and GFP difference time course. This randomised resampling step was repeated 1000 times, to generate as many GFP difference time courses. The original GFP difference at each time point within a time window of interest was then compared to the maximum GFP differences obtained within that time window over the randomisation iterations, to calculate a time point-wise t-value and p-value. Significant time points with p-values < 0.05 were clustered together based on temporal contiguity, and the cluster with the largest sum of constituent t-values, the *cluster*-*level* t-value, was retained. This procedure was then repeated for the GFP difference generated in each randomisation iteration, to identify the largest such cluster generated in each iteration. Finally, the cluster-level t-value generated with the original GFP difference was compared to the distribution of cluster-level t-values generated by the randomisation iterations, to calculate a non-parametric p-value. This represented the Monte Carlo estimate of the level of statistical significance of the cluster identified in the original GFP. As shown by Maris (2004) and Maris and Oostenveld (2007), this comparison of the original GFP difference at each time point to the *maximal* GFP difference obtained in each iteration, followed by temporal clustering of time points, effectively and sensitively controls for familywise error (FWE) and multiple comparisons. Cluster-level t-values and p-values calculated as above are reported in the text and figures.

In addition, we tested for the statistical consistency of topographical structure within a time window of interest across the individual epochs/subject-wise averages comprising an ERP, using the Topographic Consistency Test (TCT) (Koenig and Melie-Garcia, 2010). This test employed a non-parametric, GFP-based approach to estimate the significance of a single ERP topography, complementary to the clustering analysis described above. Briefly, the GFP of an ERP at each time point within a time window of interest was compared to the distribution of GFPs at the same time point, calculated over 1000 randomisation iterations in each of which the scalp topography of individual epochs was repeatedly randomised. This generated a Monte Carlo p-value that represented the probability with which the original GFP topography could have been generated just by chance (see Koenig and Melie-Garcia (2010) for details). These p-values generated by the TCT are reported in the figures, alongside results from the clustering analysis.

### 2.5 fMRI data collection and analysis

The fMRI mental imagery task first employed by Owen et al. (2006) and later replicated by Monti et al. (2010) was used to assess covert command following and volitional awareness in patients. fMRI data were collected and analysed as described by Monti et al. (2010) and later extended by Bardin et al. (2011). The key brain regions, if any, which were significantly active during tennis imagery after familywise error correction, are indicated in Table 1.

## 3 Results and discussion

We introduce our findings with the group analysis of responses elicited by healthy volunteers to explicit target, implicit target and distractor words in our experimental task. As we will show, implicit targets evoke a P3a due to their exogenous or bottom-up novelty. In contrast, explicit targets, which were as frequent as implicit targets, evoke *both* a P3a and then a P3b, due to their additional top-down or endogenous task relevance. We repeat this analysis of the P3a/b at the single-subject level with the healthy volunteer group, to investigate the robustness of the group-level effects across individual subjects. Having established the normative pattern of responses obtained with the healthy group, we move to the analysis of responses obtained in our patient population. Specifically, we focus on patients who generated discernible responses to the three types of words and evaluate these at the single-subject level.

### 3.1 Healthy volunteers: group results

**Fig. S2:**
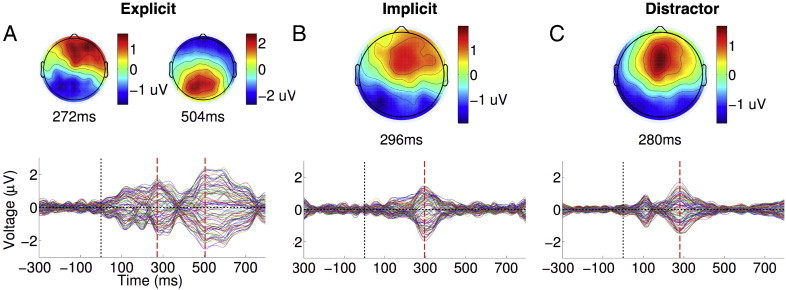
ERPs elicited by targets and distractors in healthy volunteers. In each panel, bottom half depicts a high-density ERP as a butterfly plot. Vertical dashed red lines indicate the time point of maximal amplitude across all electrodes, within the 100–400 ms P3a window or the 400–700 ms P3b window. The top half of each panel plots the topography of voltages at this maximal time point, which is specified in the text below. Panels A, B and C show the grand averaged ERPs elicited by explicit targets, implicit targets and distractors, respectively, in healthy volunteers. Explicit targets elicited both an early P3a and then a late P3b, whereas implicit targets and distractors only elicited early P3a ERPs.

Fig. 1A depicts the Global Field Power (GFP), a reference-free magnitude of electrical potential over the head (Lehmann and Skrandies, 1980; Skrandies, 1990), of the grand averaged ERP elicited by explicit targets in healthy volunteers (see Inline Supplementary Fig. S2 for the ERPs). As is evident, such targets generated a frontal P3a component, followed immediately by a parietal P3b. Here, and throughout the results described below, we statistically compared single-subject GFPs as a function of time, within the 100–400 ms P3a window and the 400–700 ms P3b window to the − 300–0 ms baseline, window using a non-parametric randomisation test (see Methods section for details). The choice of these windows were based on inclusive, normative bounds established by the time periods of statistically significant P3a/P3b clusters observed in the results of the single-subject analysis of the healthy volunteer data (see Figs. 1D and E), described in detail in the next section. The non-parametric analysis identified clusters of time points where the two ERP components were significantly larger than baseline, confirming the presence of the P3a and P3b to explicit targets at the group level. The temporal extents of the clusters obtained are indicated in Fig. 1A, along with their cluster-level t- and p-values, which indicated significant, consecutive P3a and P3b ERPs to explicit targets. To further verify the statistical reliability of these ERPs, we employed the Topographic Consistency Test (TCT) (see Koenig and Melie-Garcia (2010) and Methods section for details) to assess the significance of a particular ERP topography within the corresponding time-window. This test generated a non-parametric p-value indicating the probability with which a particular topography could be generated by chance. These TCT p-values, also indicated in Fig. 1A, were congruent with the results with the clustering analysis, and confirmed the presence of significant P3a and P3b topographies.

Fig. 1A depicts the Global Field Power (GFP), a reference-free magnitude of electrical potential over the head (Lehmann and Skrandies, 1980; Skrandies, 1990), of the grand averaged ERP elicited by explicit targets in healthy volunteers (see Inline Supplementary Fig. S2 for the ERPs). As is evident, such targets generated a frontal P3a component, followed immediately by a parietal P3b. Here, and throughout the results described below, we statistically compared single-subject GFPs as a function of time, within the 100–400 ms P3a window and the 400–700 ms P3b window to the − 300–0 ms baseline, window using a non-parametric randomisation test (see Methods section for details). The choice of these windows were based on inclusive, normative bounds established by the time periods of statistically significant P3a/P3b clusters observed in the results of the single-subject analysis of the healthy volunteer data (see Figs. 1D and E), described in detail in the next section. The non-parametric analysis identified clusters of time points where the two ERP components were significantly larger than baseline, confirming the presence of the P3a and P3b to explicit targets at the group level. The temporal extents of the clusters obtained are indicated in Fig. 1A, along with their cluster-level t- and p-values, which indicated significant, consecutive P3a and P3b ERPs to explicit targets. To further verify the statistical reliability of these ERPs, we employed the Topographic Consistency Test (TCT) (see Koenig and Melie-Garcia (2010) and Methods section for details) to assess the significance of a particular ERP topography within the corresponding time-window. This test generated a non-parametric p-value indicating the probability with which a particular topography could be generated by chance. These TCT p-values, also indicated in Fig. 1A, were congruent with the results with the clustering analysis, and confirmed the presence of significant P3a and P3b topographies.

In contrast to explicit targets, implicit targets, depicted in Fig. 1B, only generated a frontally centred P3a. A statistical comparison of the GFP of this component within the 100–400 ms window to the baseline period resulted in a significant temporal cluster. Further, a direct comparison of the GFP time courses elicited by explicit and implicit targets during the 100–400 ms P3a window produced no significant differences, but produced a significant cluster (cluster-level t = 459.84, p = 0.001) during the 400–700 ms P3b time window, demonstrating the late-stage discriminative processing elicited *only* by explicit targets. Distractor words generated a significant but relatively more focal frontocentral P3a response, as shown in Fig. 1C. The GFP of this response was statistically indistinguishable from the combined P3a component elicited by explicit and implicit targets.

Inline Supplementary Figure S2

Inline Supplementary Fig. S2 can be found online at http://dx.doi.org/10.1016/j.nicl.2013.10.008.

The substantial research literature about the cognitive processes underlying these components has established key distinctions that guided our interpretation of the pattern of results described above. We surmised that task-irrelevant implicit targets and distractors triggered early exogenous attentional orienting in response to their bottom-up, i.e., stimulus-driven novelty. But a top-down, endogenous bias towards explicit targets, set up a priori by the task instruction, meant that this bottom-up activation did not propagate any further. Explicit targets, by the virtue of their task relevance, not only triggered early exogenous attentional orienting, but also engaged later processing to imprint on conscious experience and trigger a working memory update. Overall, this pattern is broadly consistent with the literature identifying dissociations between the P3a and P3b as indexes of distinct attentional processes (Polich, 2007).

**Fig. S3:**
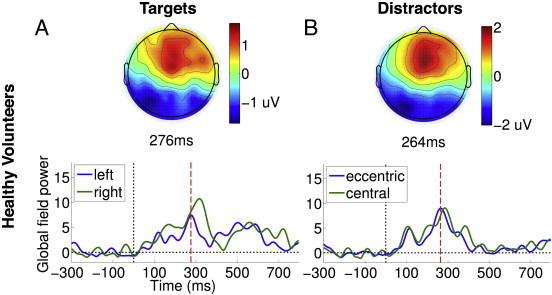
Responses to left/right-lateralised targets and eccentric/distractors in healthy volunteers. Panel A depicts the GFP time courses elicited by targets presented with apparent left and right spatial orientations. Panel B depicts the GFP time courses elicited by distractors in apparent eccentric (− 68° and + 68°) and central (0°) spatial orientations. There was no evidence of a statistical difference between either pair of GFPs.

We further tested whether participants could be deploying auditory spatial attention to the apparent orientation of targets and distractors to aid the discrimination of targets from distractors. To investigate this, we first compared the GFP time courses of eccentric (with apparent spatial orientation of − 68° and + 68°) and central distractors (presented at 0°). We hypothesised that if participants, as a group, were allocating *spatially* discriminating targets from distractors, we might expect to see differential modulation of the responses to eccentric distractors, which were much closer in spatial orientation to targets than central distractors. However, we found no reliable statistical difference in the corresponding GFP time courses (see Inline Supplementary Fig. S3B). In addition, we confirmed that there were no consistent spatial biases in the allocation of attention across the healthy volunteers, by comparing the GFP time courses of the ERPs elicited by left (− 90°) and right (+ 90°) lateralised targets (see Inline Supplementary Fig. S3A). Left and right targets both elicited a common P3a ERPs that were statistically indistinguishable: i.e., the P3b ERP elicited only by explicit targets was averaged out, leaving only the commonly elicited P3a ERP. These findings, taken together, did not provide any positive evidence in favour of a spatial attentional bias in our data. Overall, they further confirmed that it was primarily endogenous attention to the changeable *word identity* of task-relevant explicit targets that was responsible for eliciting the P3b ERP, as only a separation of trials based on word identity produced a statistical significance between the two kinds of targets, in the form of the late P3b.

We further tested whether participants could be deploying auditory spatial attention to the apparent orientation of targets and distractors to aid the discrimination of targets from distractors. To investigate this, we first compared the GFP time courses of eccentric (with apparent spatial orientation of − 68° and + 68°) and central distractors (presented at 0°). We hypothesised that if participants, as a group, were allocating *spatially* discriminating targets from distractors, we might expect to see differential modulation of the responses to eccentric distractors, which were much closer in spatial orientation to targets than central distractors. However, we found no reliable statistical difference in the corresponding GFP time courses (see Inline Supplementary Fig. S3B). In addition, we confirmed that there were no consistent spatial biases in the allocation of attention across the healthy volunteers, by comparing the GFP time courses of the ERPs elicited by left (− 90°) and right (+ 90°) lateralised targets (see Inline Supplementary Fig. S3A). Left and right targets both elicited a common P3a ERPs that were statistically indistinguishable: i.e., the P3b ERP elicited only by explicit targets was averaged out, leaving only the commonly elicited P3a ERP. These findings, taken together, did not provide any positive evidence in favour of a spatial attentional bias in our data. Overall, they further confirmed that it was primarily endogenous attention to the changeable *word identity* of task-relevant explicit targets that was responsible for eliciting the P3b ERP, as only a separation of trials based on word identity produced a statistical significance between the two kinds of targets, in the form of the late P3b.

Inline Supplementary Figure S3

Inline Supplementary Fig. S3 can be found online at http://dx.doi.org/10.1016/j.nicl.2013.10.008.

### 3.2 Healthy volunteers: individual results

We tested the discriminability of the P300 responses elicited by targets and distractors at a single-subject level, to ascertain the extent to which the group-level results described above could be extrapolated to interpret results from individual subjects. Fig. 1D plots the statistical significance of GFP elicited by explicit targets in each healthy volunteer as a stacked image, allowing us to visualise the pattern of single-subject statistics across the group. As is evident, this pattern broadly corresponds to that observed at the group level (Fig. 1A): In all healthy volunteers, explicit targets elicited a consistently significant P3b in the 400–700 ms window, and a P3a within the 100–400 ms in five of them. Single-subject statistics of responses to implicit targets and distractors, depicted as colour maps in Figs. 1E and F, had a similar relationship to the group-level pattern (Figs. 1B and C, respectively): both evoked a reliable early P3a response in all subjects.

### 3.3 Patients: group results

We analysed data from VS and MCS patients with the aim of dissociating the P3a and P3b in such states of clinically impaired consciousness. In doing so, we hoped to establish whether the distinct bottom-up/top-down attentional engagement these ERP components reflect could be separably elicited in a patient.

Of the 30 patients included in the study, data from 9 patients could not be analysed due to excessive levels of artefact. Table 1 lists demographic information about the 21 patients whose EEG data was analysed, along with their behavioural score on the Coma Recovery Scale—Revised (CRS-R) (Kalmar and Giacino, 2005) and consequent diagnosis (VS or MCS). Individual scores measured with each patient on the CRS-R subscales are listed in Table 2. These 21 patients also participated in the fMRI tennis imagery task, previously used to assess volitional command following and covert awareness in DoC (Bardin et al., 2011; Monti et al., 2010; Owen et al., 2006). Of the 21 patients, fMRI data from one MCS patient could not be analysed due to excessive artefact. As listed in Table 1, of the remaining 20 patients (9 VS and 11 MCS), 9 produced significant BOLD activations in the tennis imagery task (4 VS and 5 MCS). These proportions were not significantly different in a Fisher's exact test (p = 0.32).

**Fig. 2:**
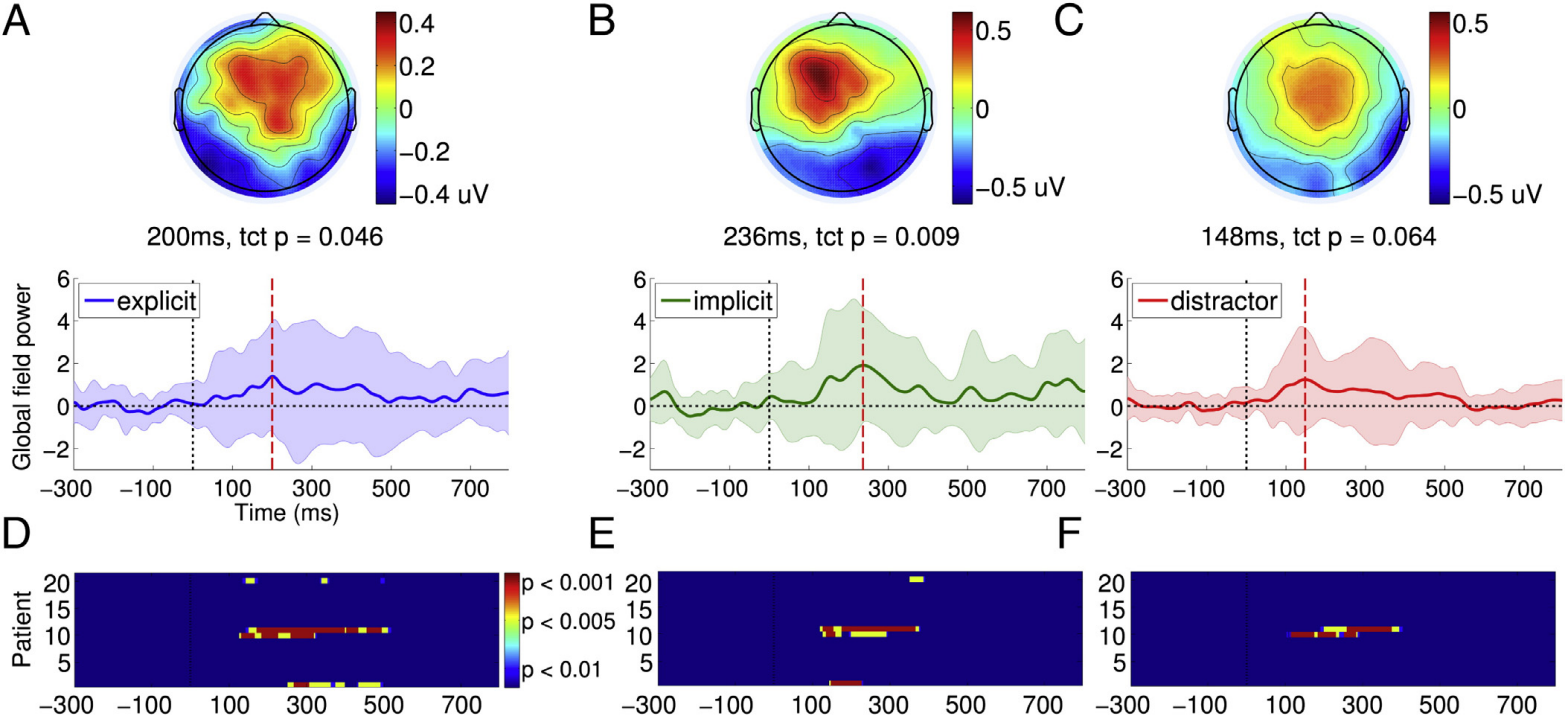
Responses to targets and distractors in patients. Explicit targets (panel A), implicit targets (panel B) and distractors (panel C) elicited qualitatively similar frontal P3a responses within the 100–400 ms in patients, with pronounced variability within the group. Panels D–F plot stacked colour maps of statistically significant clusters of GFP observed in individual patients. Each horizontal line plots the significant time course of an individual patient's GFP on a colour scale. Panel D shows individual GFP clusters within the 100–400 ms P3a window and the 400–700 ms P3b window for explicit targets. Similarly, panels E and F show clusters within the 100–400 ms window for implicit targets and distractors, respectively.

Figs. 2A–C plot the GFP time courses of ERPs elicited by targets and distractors in the EEG task, averaged over all the patients. When compared to corresponding activations in healthy volunteers (Figs. 1A-C), the similarities and differences are evident: on average, patients generated weak P3a-like frontally centred responses to both kinds of targets within the 100-400 ms window, which were not significantly above baseline in a group-level analysis. Further, in contrast to healthy volunteers, there was no evidence of endogenous processing that discriminated explicit and implicit targets in the later 400-700 ms P3b window.

### 3.4 Patients: Individual Results

Fig. 2D–F depicts stacked colour maps of significant temporal clusters within GFP time courses of ERPs elicited by individual patients. These were calculated by comparing the GFP of each patient ERP within the 100–400 ms P3a or the 400–700 ms P3b window to the − 300–0 ms baseline, using the same procedure as that used for single-subject statistical analysis of data from healthy volunteers. In contrast to the corresponding maps in healthy volunteers (Fig. 1D–F), there was a prominent but expected inconsistency in the responses across the patient group. As is evident, in all but 4 patients, targets and distractors did not generate statistically significant ERP responses, explaining the lack of a significant response at the group level in the previous section. In these four patients (from a group of 9 VS and 12 MCS), a heterogeneous pattern of responses was observed. As shown in Table 1, one of these patients was VS (P1) and three (P10, P11 and P20) were MCS (proportions were not significantly different: Fisher's exact p = 0.33). Three of them had suffered traumatic injury (P1, P10 and P20) and one (P11) anoxic injury (Fisher's exact p = 0.45). Importantly however, three out of these four patients also generated independent fMRI evidence of tennis imagery. Delving further, we investigated the presence of P3a and P3b responses to implicit and explicit targets in these patients, who are discussed below individually.

#### 3.4.1 Patient P1

**Fig. S4:**
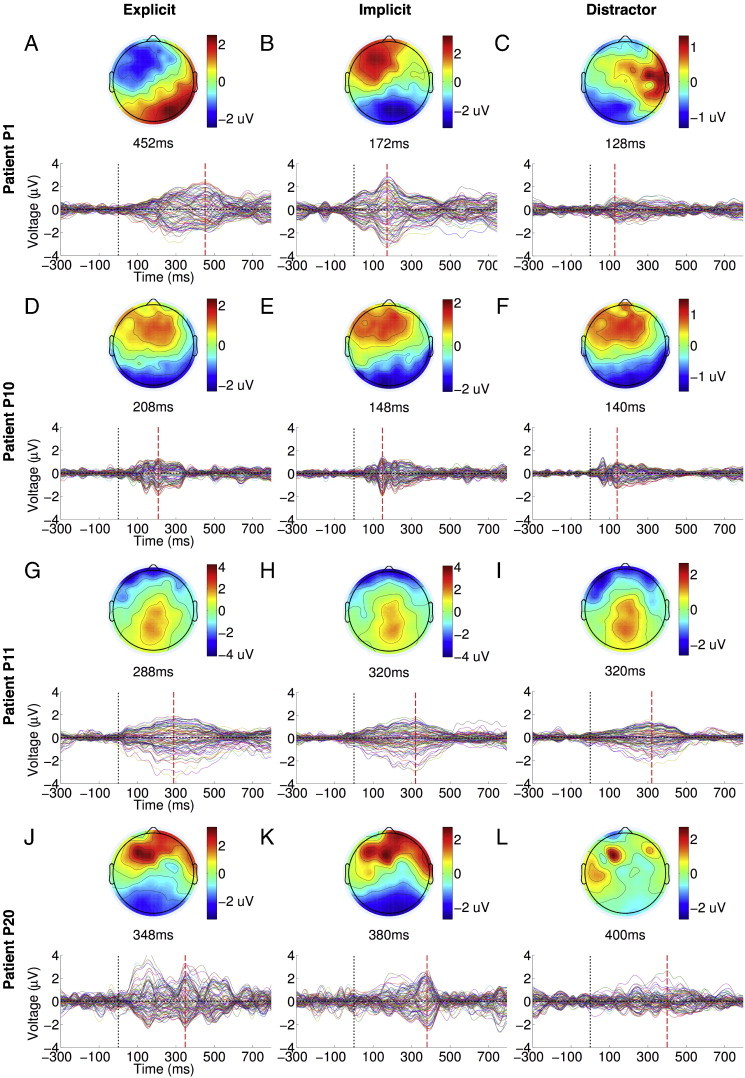
ERPs elicited by targets and distractors in patients. Panels A–C, D–F, G–I and J–L depict the ERPs elicited by explicit targets, implicit targets and distractors, in patients P1, P10, P11 and P20, respectively. In P1, explicit targets elicited a late P3b, whereas implicit targets elicited an early P3a. In patients P10, P11 and P20, explicit and implicit targets only elicited early responses.

**Fig. 3:**
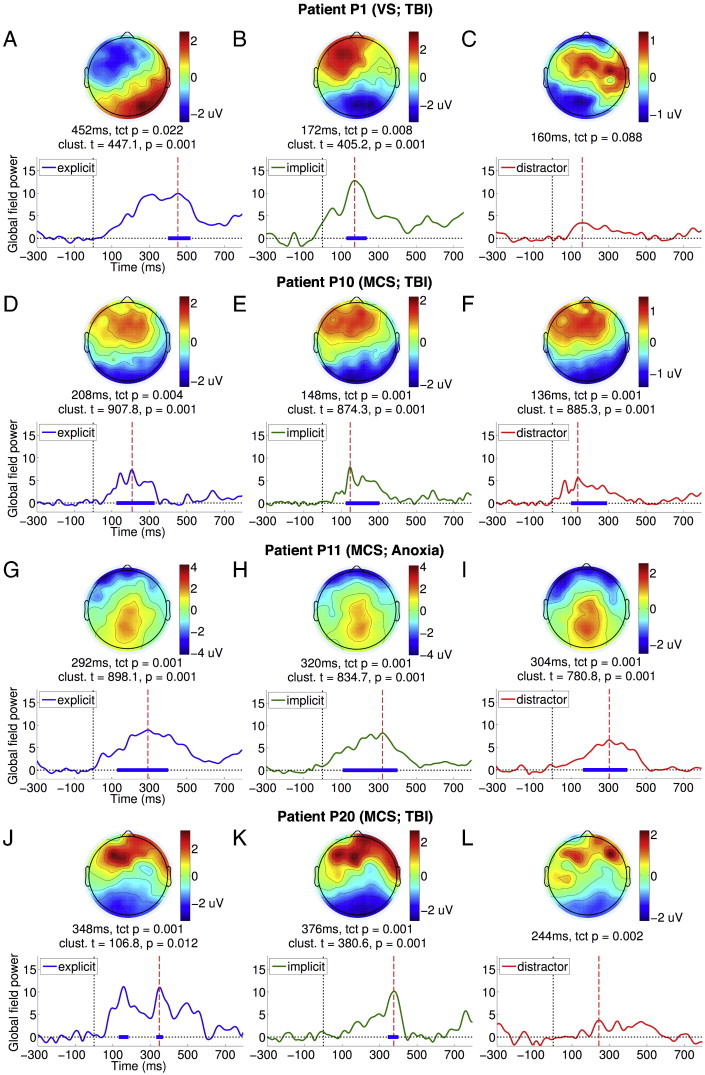
Responses in patients P1, P10, P11 and P20. Panels A–C, D–F, G–I and J–L plot GFP time courses, significant clusters and scalp topographies of responses to explicit targets, implicit targets and distractors for patients P1, P10, P11 and P20, respectively. In P1, explicit targets elicited a significant parietal P3b within the 400–700 ms window (panel A), whereas implicit targets generated a frontal P3a within the 100–400 ms window (panel B). In P10, both explicit and implicit targets similar frontal P3a responses that were significant only within the 100–400 ms window (panels D and E). Similarly, P11 generated temporally extended parietally focused responses that were common to both explicit and implicit targets (panels G and H). Finally, P20 generated frontal P3a responses to both types of targets, which were significantly greater than the response to distractors within the 100–400 ms window (panels J and K).

P1 suffered traumatic brain injury 4 months prior to testing, and appeared to be behaviourally vegetative, based on a maximum CRS-R score of 7. Based on the CRS-R assessment, there was no evidence of command following at the bedside (see Table 2). Fig. 3A plots the GFP time course of the ERP elicited by explicit targets presented to P1 (see Inline Supplementary Fig. S4 for the ERPs). As can be seen, there was a sustained late response between the 200 and 500 ms window, which peaked at 452 ms. The parietally focused positivity of the scalp topography at this peak was suggestive of a P3b response. We statistically compared GFP during the 400–700 ms window to the − 300–0 ms baseline using a non-parametric randomisation test, identical to that used to test single-subject ERPs from healthy volunteers (Fig. 1D–F). This comparison produced a strongly significant temporal cluster, confirming the significant parietal P3b elicited by explicit targets. In addition, the TCT p-value was significant, indicating the presence of a P3b topography that was statistically consistent across the individual trials making up the ERP. Furthermore, there was a significant channel-wise correlation between the peak topography of P1's P3b (Fig. 3A, top) and the average P3b topography in the healthy volunteer group (Fig. 1A, top right): Pearson's r = 0.57, p < 0.0001, confirming that the patient's P3b conformed topographically to the normative pattern established in the healthy volunteers.

P1 suffered traumatic brain injury 4 months prior to testing, and appeared to be behaviourally vegetative, based on a maximum CRS-R score of 7. Based on the CRS-R assessment, there was no evidence of command following at the bedside (see Table 2). Fig. 3A plots the GFP time course of the ERP elicited by explicit targets presented to P1 (see Inline Supplementary Fig. S4 for the ERPs). As can be seen, there was a sustained late response between the 200 and 500 ms window, which peaked at 452 ms. The parietally focused positivity of the scalp topography at this peak was suggestive of a P3b response. We statistically compared GFP during the 400–700 ms window to the − 300–0 ms baseline using a non-parametric randomisation test, identical to that used to test single-subject ERPs from healthy volunteers (Fig. 1D–F). This comparison produced a strongly significant temporal cluster, confirming the significant parietal P3b elicited by explicit targets. In addition, the TCT p-value was significant, indicating the presence of a P3b topography that was statistically consistent across the individual trials making up the ERP. Furthermore, there was a significant channel-wise correlation between the peak topography of P1's P3b (Fig. 3A, top) and the average P3b topography in the healthy volunteer group (Fig. 1A, top right): Pearson's r = 0.57, p < 0.0001, confirming that the patient's P3b conformed topographically to the normative pattern established in the healthy volunteers.

In contrast, implicit targets evoked a markedly different and relatively early frontal response shown in Fig. 3B. The GFP of this response was statistically significant relative to baseline and generated a significant TCT p-value within the 100–400 ms window, suggesting the presence of a consistent P3a to implicit targets, though this response was abnormally early in comparison to healthy adults (Fig. 1B). Nevertheless, its peak topography (Fig. 3B, top) was highly significantly correlated with the normative P3a topography in healthy volunteers (Fig. 1B, top): Pearson's r = 0.73, p < 0.0001.

**Fig. S5:**
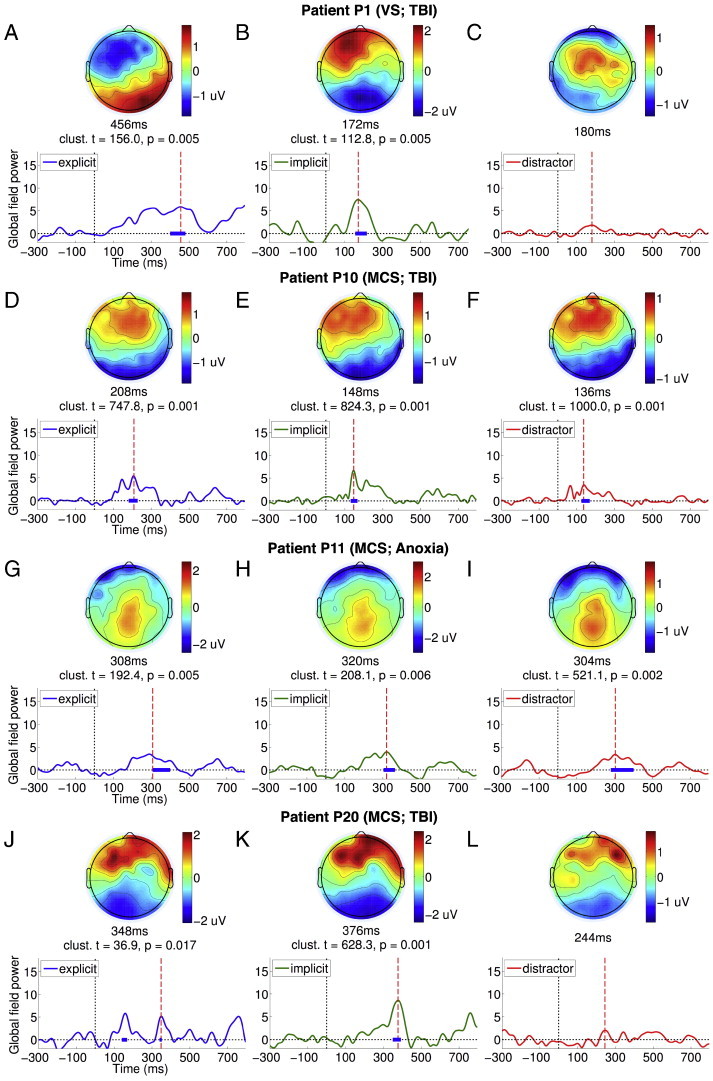
Responses in patients P1, P10, P11 and P20 after baseline correction to the whole epoch. Panels A–C, D–F, G–I and J–L plot GFP time courses, significant clusters and scalp topographies of responses to explicit targets, implicit targets and distractors for patients P1, P10, P11 and P20, respectively, after baseline correction to the whole epoch from − 300 to 800 ms. There were no qualitative changes in the pattern of effects observed, relative to the same comparisons after baseline correction to − 300–0 ms, depicted in Fig. 3.

We ensured that the size of these significant GFP effects observed in P1, involving a comparison to baseline, was not artificially inflated by the baseline correction step applied during the pre-processing of the EEG data. To verify this, we repeated the same statistical analysis as above, but after applying the baseline correction to the *entire* epoch from − 300 to 800 ms. The results of this reanalysis are shown in Inline Supplementary Fig. S5, which depicts exactly the same GFP comparisons as in Fig. 3, but with the whole-epoch baseline correction. As can be seen by comparing the two figures, the significant responses elicited in P1 persisted with the whole-epoch baseline. In fact, there was no qualitative change in the pattern of effects observed in any of the other patients discussed later in this section.

We ensured that the size of these significant GFP effects observed in P1, involving a comparison to baseline, was not artificially inflated by the baseline correction step applied during the pre-processing of the EEG data. To verify this, we repeated the same statistical analysis as above, but after applying the baseline correction to the *entire* epoch from − 300 to 800 ms. The results of this reanalysis are shown in Inline Supplementary Fig. S5, which depicts exactly the same GFP comparisons as in Fig. 3, but with the whole-epoch baseline correction. As can be seen by comparing the two figures, the significant responses elicited in P1 persisted with the whole-epoch baseline. In fact, there was no qualitative change in the pattern of effects observed in any of the other patients discussed later in this section.

**Fig. S6:**
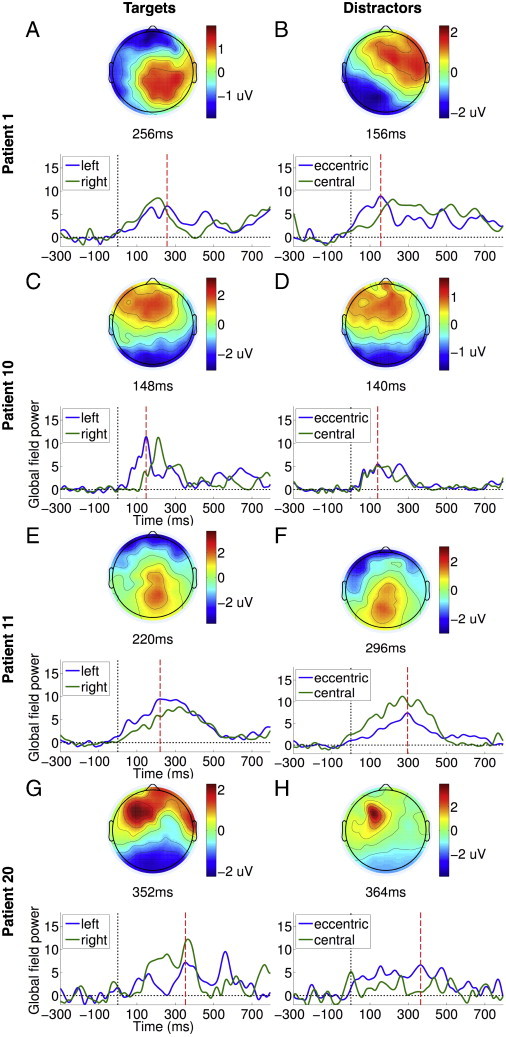
Responses to left/right-lateralised targets and eccentric/distractors in patients. Panels A–B, C–D, E–F and G–H depict the GFP time courses elicited by left/right-lateralised targets and eccentric/central distractors, in patients P1, P10, P11 and P20, respectively. There was no evidence of a statistical difference between any pair of GFPs.

Going further, we directly compared responses to explicit and implicit targets shown in Fig. 3A and B within the 400–700 ms window, which resulted in a significant cluster (cluster-level t = 199.34, p = 0.01). This confirmed that *only* explicit targets elicited P3b. Finally, we found that distractors generated a similarly early, weak frontocentral response (Fig. 3C). However, the GFP of this response was *not* statistically higher than baseline levels of activation. Following up this result, we compared GFPs of responses to implicit targets and distractors within the early 100–400 ms P3a window. The presence of a significant cluster (cluster-level t = 179.38, p = 0.01) indicated that P1 was orienting attention to targets in response to their greater bottom-up salience than distractors. Interestingly, we did not observe the same difference between implicit targets and distractors in healthy volunteers (see Fig. 1B and C). Finally, we noted that, like in healthy volunteers, there was no statistically significant difference observable in the GFP time courses elicited by left/right lateralised targets or eccentric/central distractors (see Inline Supplementary Fig. S6A and B), suggesting that there was no spatial differentiation or laterality-specific deficit observable in allocation of attention by P1.

Going further, we directly compared responses to explicit and implicit targets shown in Fig. 3A and B within the 400–700 ms window, which resulted in a significant cluster (cluster-level t = 199.34, p = 0.01). This confirmed that *only* explicit targets elicited P3b. Finally, we found that distractors generated a similarly early, weak frontocentral response (Fig. 3C). However, the GFP of this response was *not* statistically higher than baseline levels of activation. Following up this result, we compared GFPs of responses to implicit targets and distractors within the early 100–400 ms P3a window. The presence of a significant cluster (cluster-level t = 179.38, p = 0.01) indicated that P1 was orienting attention to targets in response to their greater bottom-up salience than distractors. Interestingly, we did not observe the same difference between implicit targets and distractors in healthy volunteers (see Fig. 1B and C). Finally, we noted that, like in healthy volunteers, there was no statistically significant difference observable in the GFP time courses elicited by left/right lateralised targets or eccentric/central distractors (see Inline Supplementary Fig. S6A and B), suggesting that there was no spatial differentiation or laterality-specific deficit observable in allocation of attention by P1.

**Fig. 4:**
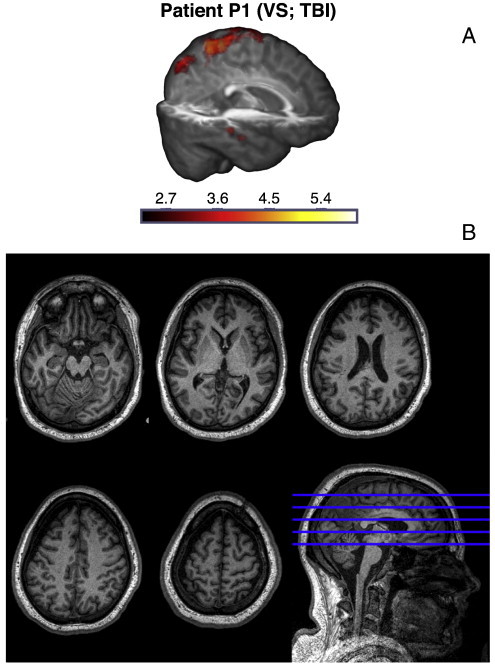
Activation in patient P1 during tennis imagery in fMRI. Panel A highlights significant activations observed in brain regions including the Supplementary Motor Area (SMA) in P1, during the tennis imagery task in fMRI. Colour scale indicates z-scores of activations. Data were collected and analysed as described in Monti et al. (2010) and Bardin et al. (2011). Panel B shows selected axial slices of P1's T1-weight MRI in native space.

On the whole, we found that P1 generated remarkable discriminative EEG responses to explicit and implicit targets that were mostly consistent with that obtained with healthy adults: an early frontal P3a for implicit targets, and a late posterior parietal P3b for explicit targets. The statistical significance of these responses, specified in Fig. 3A and B, were robust enough to survive a Bonferroni–Holm (Holm, 1979) correction for multiple comparisons across the group of 21 patients. Hence there was strong evidence that P1 was able to follow auditory instructions that varied by block to set up top-down attentional bias in favour of task-relevant explicit targets, while also orienting to exogenously salient but task-irrelevant implicit targets. Furthermore, the patient also generated significant activation in the Supplementary Motor Area (SMA), as measured by fMRI, when following commands to perform tennis imagery (see Fig. 4A). This area has previously been shown to be active when healthy volunteers and patients were asked to perform such motor imaginations (Bardin et al., 2011; Monti et al., 2010; Owen et al., 2006). This fMRI evidence of P1's volitional abilities independently corroborated our EEG findings, and made a persuasive case for the presence of covert awareness in the behaviourally vegetative state. Finally, P1 also had a relatively low cortical atrophy score of 1 (see Table 1). As can be seen in the T1-weighted images (see Fig. 4B), we observed cortical damage particularly in left frontal regions, but otherwise relatively preserved neural integrity, perhaps providing the necessary substrate for generating P3b responses. In the Results and discussion section, we consider the implications of these findings for our understanding of attention and consciousness in states of impaired consciousness. Next, we highlight three additional patients whose responses present a contrasting picture.

#### 3.4.2 Patient P10

P10 was a minimally conscious patient (maximum CRS-R score = 10), tested 68 months after traumatic brain injury. We observed evidence of command following at the bedside during CRS-R assessment. Fig. 3D and E plots GFPs of the responses to explicit and implicit targets elicited (see Inline Supplementary Fig. S4 for the ERPs) in the patient. P10 produced clear early frontal P3a responses to explicit and implicit targets, GFPs of which were significant within the 100–400 ms relative to the − 300–0 ms baseline, and generated significant TCT p-values. In addition, the peak topographies of these P3a responses were significantly channel-wise correlated to those observed in healthy volunteers (explicit: Pearson's r = 0.74, p < 0.0001; implicit: Pearson's r = 0.8 p < 0.0001). However, there was no significant difference between these early responses to explicit and implicit targets in a direct comparison. In addition, there was no evidence of a later response to explicit targets within the P3b time window. Further, there were no statistically significant differences observed in a direct comparison of explicit and implicit targets. Taken together, this pattern suggested that both types of targets evoked similar responses. In addition, distractors generated a similarly significant response (Fig. 3F), the GFP of which was statistically indistinguishable from that of either explicit or implicit targets within the 100–400 ms P3a time window. Hence there was no evidence that the activation generated in response to targets was sensitive to their differential bottom-up novelty relative to distractors. Interestingly, we noted that there was a suggestion of a latency difference in the GFPs of the ERPs elicited by left and right targets (see Inline Supplementary Fig. S6C), though the peak amplitudes were similar, suggesting a lateralised deficit in the temporal dynamics of attention and perception in P10. However, we did not observe any such lateralisation effect when comparing eccentric vs. central distractors (Inline Supplementary Fig. S6D).

P10 was a minimally conscious patient (maximum CRS-R score = 10), tested 68 months after traumatic brain injury. We observed evidence of command following at the bedside during CRS-R assessment. Fig. 3D and E plots GFPs of the responses to explicit and implicit targets elicited (see Inline Supplementary Fig. S4 for the ERPs) in the patient. P10 produced clear early frontal P3a responses to explicit and implicit targets, GFPs of which were significant within the 100–400 ms relative to the − 300–0 ms baseline, and generated significant TCT p-values. In addition, the peak topographies of these P3a responses were significantly channel-wise correlated to those observed in healthy volunteers (explicit: Pearson's r = 0.74, p < 0.0001; implicit: Pearson's r = 0.8 p < 0.0001). However, there was no significant difference between these early responses to explicit and implicit targets in a direct comparison. In addition, there was no evidence of a later response to explicit targets within the P3b time window. Further, there were no statistically significant differences observed in a direct comparison of explicit and implicit targets. Taken together, this pattern suggested that both types of targets evoked similar responses. In addition, distractors generated a similarly significant response (Fig. 3F), the GFP of which was statistically indistinguishable from that of either explicit or implicit targets within the 100–400 ms P3a time window. Hence there was no evidence that the activation generated in response to targets was sensitive to their differential bottom-up novelty relative to distractors. Interestingly, we noted that there was a suggestion of a latency difference in the GFPs of the ERPs elicited by left and right targets (see Inline Supplementary Fig. S6C), though the peak amplitudes were similar, suggesting a lateralised deficit in the temporal dynamics of attention and perception in P10. However, we did not observe any such lateralisation effect when comparing eccentric vs. central distractors (Inline Supplementary Fig. S6D).

Inline Supplementary Figure S4

Inline Supplementary Fig. S4 can be found online at http://dx.doi.org/10.1016/j.nicl.2013.10.008.

Overall, P10 presented a different pattern of ERPs when compared to P1: there was evidence of exogenous, stimulus-triggered attentional orienting to targets of both kinds. But crucially, the response did not discriminate between the two types of targets: that is, the early response to explicit targets was not followed up by engagement of higher-order perception and working memory processes. This could have been due to P10's inability to comprehend or remember the task instructions and appropriately deploy top-down attention to explicit targets. Alternatively, such endogenous attentional control, even if present, might have been too inconsistent or too weak to produce further processing indexed by the P3b. Indeed, the substantially greater level of arousal variation, and perhaps post-traumatic confusion (Nakase-Richardson et al., 2009), observed in MCS patients is consistent with the latter interpretation. In keeping with this, we observed significant variation in P10's behaviour, indexed by a range of CRS-R scores between 5 (VS) and 10 (MCS) measured over four days of observation. In addition, we did not find any evidence of command following in P10 with the fMRI tennis imagery task, and measured a relatively high cortical atrophy score of 3.

#### 3.4.3 Patient P11

P11 was tested 6 months after anoxic brain injury, and was diagnosed as minimally conscious (maximum CRS-R score = 9) at the time. Based on the CRS-R assessment, there was no evidence of command following at the bedside. Fig. 3G and H plot GFP time courses of the patient's ERP responses to explicit and implicit targets (see Inline Supplementary Fig. S4 for the ERPs). Fig. 3I plots the response to distractors. The responses were all temporally diffuse and sustained, and shared a parietally focused topographical locus. A statistical comparison of the GFPs between 100 and 400 ms relative to the − 300 and 0 ms baseline, along with their TCT p-values, revealed significant clusters in all 3 conditions, as shown in the figures. However, there was no positive correlation between the peak topographies in P11 and in healthy volunteers within the P3a time window. In a direct comparison, we found that there was no significant difference in GFP between explicit and implicit targets. Furthermore, GFP of responses to both kinds of targets were statistically indistinguishable from distractors. Alongside, there was no significant effect of spatial orientation of stimuli, either of targets or distractors (see Inline Supplementary Fig. S6E and F).

P11 was tested 6 months after anoxic brain injury, and was diagnosed as minimally conscious (maximum CRS-R score = 9) at the time. Based on the CRS-R assessment, there was no evidence of command following at the bedside. Fig. 3G and H plot GFP time courses of the patient's ERP responses to explicit and implicit targets (see Inline Supplementary Fig. S4 for the ERPs). Fig. 3I plots the response to distractors. The responses were all temporally diffuse and sustained, and shared a parietally focused topographical locus. A statistical comparison of the GFPs between 100 and 400 ms relative to the − 300 and 0 ms baseline, along with their TCT p-values, revealed significant clusters in all 3 conditions, as shown in the figures. However, there was no positive correlation between the peak topographies in P11 and in healthy volunteers within the P3a time window. In a direct comparison, we found that there was no significant difference in GFP between explicit and implicit targets. Furthermore, GFP of responses to both kinds of targets were statistically indistinguishable from distractors. Alongside, there was no significant effect of spatial orientation of stimuli, either of targets or distractors (see Inline Supplementary Fig. S6E and F).

P11's responses present an interesting disjunction to the previous two patients. Though the ERPs had a significant parietal locus, we could not conclude that this was evidence of a P3b ERP component, as the response was indiscriminate, both across conditions and across time. Like the pattern with P10's responses, explicit and implicit targets evoked statistically indistinguishable activations, suggesting a lack of any discriminative processing. Hence, though there was evidence of exogenous, stimulus-triggered attentional orienting to targets, explicit targets did not benefit from top-down task-contingent attention. However, P11 did generate evidence of command following with fMRI: we observed significant activation in the parietal cortex, specifically in the Intraparietal Sulcus (IPS), during tennis imagery. This finding is consistent with previous findings of increased parietal cortex activity during mental imagery in DoC patients (Bardin et al., 2011), suggesting some degree of volitional awareness in P11, at least during the fMRI task. Alongside, P11's cortical atrophy score of 2 indicated a relatively mild level of neural degeneration.

Inline Supplementary Figure S5

Inline Supplementary Fig. S5 can be found online at http://dx.doi.org/10.1016/j.nicl.2013.10.008.

#### 3.4.4 Patient P20

P20 was a minimally conscious patient with a relatively high CRS-R score of 18, tested 10 months after traumatic brain injury. We observed evidence of command following at the bedside during CRS-R assessment. Fig. 3J and K plots GFPs of the responses to explicit and implicit targets, respectively (see Inline Supplementary Fig. S4 for the ERPs). P20 generated a frontally centred positivity within the 100–400 ms P3a time window to both types of targets, the GFPs of which reached levels significantly above the − 300–0 ms baseline and had significant TCT p-values. The peak topographies of these responses were significantly channel-wise correlated to those observed in healthy volunteers (explicit: Pearson's r = 0.77, p < 0.0001; implicit: Pearson's r = 0.76 p < 0.0001). However, there was no evidence of a late response to explicit targets within the P3b time window, and no significant difference between the responses to explicit and implicit targets in a direct comparison. However, both types of targets taken together generated a significantly larger GFP response than that generated by distractors (Fig. 3L) within the 100–400 ms window (cluster-level t = 182.81, p = 0.009), suggesting that targets in general generated larger responses due to their differential frequency of occurrence. However, like with patients P10 and P11, explicit targets did not seem to benefit from top-down enhancement that is sensitive to their endogenous task relevance. In addition, there was no evidence of spatial orientation bias or deficit evident in GFPs elicited by left/right targets or eccentric/central distractors (see Inline Supplementary Fig. S6G and H).

P20 was a minimally conscious patient with a relatively high CRS-R score of 18, tested 10 months after traumatic brain injury. We observed evidence of command following at the bedside during CRS-R assessment. Fig. 3J and K plots GFPs of the responses to explicit and implicit targets, respectively (see Inline Supplementary Fig. S4 for the ERPs). P20 generated a frontally centred positivity within the 100–400 ms P3a time window to both types of targets, the GFPs of which reached levels significantly above the − 300–0 ms baseline and had significant TCT p-values. The peak topographies of these responses were significantly channel-wise correlated to those observed in healthy volunteers (explicit: Pearson's r = 0.77, p < 0.0001; implicit: Pearson's r = 0.76 p < 0.0001). However, there was no evidence of a late response to explicit targets within the P3b time window, and no significant difference between the responses to explicit and implicit targets in a direct comparison. However, both types of targets taken together generated a significantly larger GFP response than that generated by distractors (Fig. 3L) within the 100–400 ms window (cluster-level t = 182.81, p = 0.009), suggesting that targets in general generated larger responses due to their differential frequency of occurrence. However, like with patients P10 and P11, explicit targets did not seem to benefit from top-down enhancement that is sensitive to their endogenous task relevance. In addition, there was no evidence of spatial orientation bias or deficit evident in GFPs elicited by left/right targets or eccentric/central distractors (see Inline Supplementary Fig. S6G and H).

Despite the lack of evidence for selective attention in EEG, P20 did generate evidence of command following with fMRI: we observed significant activation in the SMA during tennis imagery. Indeed, like P11, who also followed command with fMRI, P20 had relatively mild cortical atrophy, with a score of 2.

Inline Supplementary Figure S6

Inline Supplementary Fig. S6 can be found online at http://dx.doi.org/10.1016/j.nicl.2013.10.008.

### 3.5 General discussion

Our results have described ERP manifestations of exogenous orienting and endogenous control of attention to auditory word stimuli in DoC patients. Employing GFP-based non-parametric statistical analysis of single-subject ERPs, we observed dissociable P3a and P3b markers of distinct attentional processes in one patient (P1) whose CRS-R scores over four days of observation, which included the days of the EEG and fMRI tests, indicated a behavioural diagnosis of vegetative state. The striking pattern of EEG activations produced by P1 belies the presence of relatively advanced attentional processing that we know to result in conscious perception in healthy adults (Bekinschtein et al., 2009b; Chennu et al., 2013; Polich, 2007). It is worth considering that the cognitive capabilities required the patient to generate early P3a and late P3b responses consistently enough to manifest significantly in ERPs. The experimental task required binaural hearing, language comprehension, short-term memory, and attentional switching. Though EEG data from patients can suffer some higher levels of noise due to motion and muscle artefacts, these are unlikely to explain away the observed responses. Data obtained from P1, who did not exhibit much physical movements at the time of recording, was relatively clean and required no ICA-based artefact rejection (see Inline Supplementary Table S2). Furthermore, purely automatic or pre-attentional neural responses to target words are also unlikely to fully account for P1's ERPs, as the mapping of target type (explicit/implicit) to word identity (‘YES’/‘NO’) was counterbalanced across blocks. Hence the experimental design ensured that only the correct block-level association between word identity and target would result in the generation of dissociable P3a and P3b ERPs. Furthermore, the presence of volitional control in P1 despite a CRS-R diagnosis of VS was independently corroborated by an fMRI test of command following, and visual evidence of a cortex with relatively high structural integrity, lending additional weight to the attentional dissociation evidenced in the patient's ERPs.

Our results have described ERP manifestations of exogenous orienting and endogenous control of attention to auditory word stimuli in DoC patients. Employing GFP-based non-parametric statistical analysis of single-subject ERPs, we observed dissociable P3a and P3b markers of distinct attentional processes in one patient (P1) whose CRS-R scores over four days of observation, which included the days of the EEG and fMRI tests, indicated a behavioural diagnosis of vegetative state. The striking pattern of EEG activations produced by P1 belies the presence of relatively advanced attentional processing that we know to result in conscious perception in healthy adults (Bekinschtein et al., 2009b; Chennu et al., 2013; Polich, 2007). It is worth considering that the cognitive capabilities required the patient to generate early P3a and late P3b responses consistently enough to manifest significantly in ERPs. The experimental task required binaural hearing, language comprehension, short-term memory, and attentional switching. Though EEG data from patients can suffer some higher levels of noise due to motion and muscle artefacts, these are unlikely to explain away the observed responses. Data obtained from P1, who did not exhibit much physical movements at the time of recording, was relatively clean and required no ICA-based artefact rejection (see Inline Supplementary Table S2). Furthermore, purely automatic or pre-attentional neural responses to target words are also unlikely to fully account for P1's ERPs, as the mapping of target type (explicit/implicit) to word identity (‘YES’/‘NO’) was counterbalanced across blocks. Hence the experimental design ensured that only the correct block-level association between word identity and target would result in the generation of dissociable P3a and P3b ERPs. Furthermore, the presence of volitional control in P1 despite a CRS-R diagnosis of VS was independently corroborated by an fMRI test of command following, and visual evidence of a cortex with relatively high structural integrity, lending additional weight to the attentional dissociation evidenced in the patient's ERPs.

The level of difficulty entailed by our attention task also explains why no patients except one were able to generate such dissociable responses. Three other patients (P10, P11 and P20) generated early non-discriminative responses to targets, suggesting that involuntary bottom-up attentional orienting might be preserved in a greater proportion of patients. In making this distinction within our cohort, our experimental design enabled us to distinguish between patients along a hierarchy of progressively complex attentional capabilities. Though previous research has shown that P300 ERPs can be generated even in VS (Faugeras et al., 2012; Kotchoubey et al., 2001), we have been able to demonstrate dissociated P3a/P3b responses independent of stimulus identity in this state. Going further, we found that the link between the presence of intermediate levels of attentional abilities and the detection of covert volitional control with fMRI was complex. Of the three patients P10, P11 and P20, all of who were MCS, two (P11 and P20) generated evidence of command following in the tennis imagery task. The considerable variability in arousal observed in MCS could speculatively explain this pattern. However, as is evident from Table 1, six patients in whom no discernible P3a/P3b ERPs could be elicited did in fact generate significant activation during the fMRI tennis imagery task (P6, P12, P15, P16, P17 and P19). Further, of the seven patients who showed evidence of bedside command following with CRS-R assessment, significant fMRI imagery activation was only observed in four, and P3a ERPs in only two patients (see Table 1). None of the patients who showed evidence of bedside command following generated a significant P3b. While this discordance could similarly be attributed to variation in arousal, fundamental differences in signal detectability in behaviour, fMRI and EEG complicate comparisons across these measurement modalities. Yet another inconsistency observed in patient EEG responses relates to the abnormality of their temporal dynamics: In patients P1 and P10, though the frontal topography of the P3a was in line with the healthy volunteer group, it peaked earlier than normal. This somewhat counterintuitive speed-up of the ERP latency remains to be explored in greater depth. While the latency of the frontal response to implicit targets in these two patients was somewhat earlier than the normative bounds of the P3a commonly identified in group analyses of healthy volunteers and should hence be interpreted with caution, early P3a latencies can in fact be observed in single-subject analyses of healthy volunteer data (see Fig. 1D and E). In P10's case, the indiscriminate nature of the ERPs generated by targets and distractors suggests that it might be indexing a non-selective orienting triggered by any stimulation.

Taken together, though our findings are *indicative* of a degree of awareness in DoC, it can be argued that they are not *demonstrative* as such (Shea and Bayne, 2010). Indeed, building a neuroscientific basis for demonstrative claims of consciousness is likely to require a collective interpretation drawing upon multiple data sources from passive and active experimental paradigms (Boly and Seth, 2012). Furthermore, however might compelling our reverse inference of P1's *level* of conscious awareness might be, elucidating what *content* the patient might actually be conscious of in tasks like ours remains challenging (Overgaard and Overgaard, 2010), involving complimentary advances in experimental designs and models of impaired consciousness (Chennu and Bekinschtein, 2012).

## 4 Conclusions

We have drawn upon the extensive literature on EEG markers of bottom-up and top-down attention, the P3a and P3b, to investigate these processes in patients affected by disorders of consciousness. Though most of the patients tested were unable to respond behaviourally in a consistent manner, we found early markers of exogenous or bottom-up attentional orienting to salient words in three of them. In particular, in one patient who fit the behavioural criteria for the vegetative state, we additionally elicited a late response indicative of endogenous, top-down attentional control. This patient also showed independent evidence of covert volitional awareness with fMRI, by performing motor imagery on command. Our findings present a persuasive case for the presence of dissociable attentional processing in patients, with implications for our understanding of levels and forms of conscious awareness of which they might be capable.

## Conflict of interest

The authors declare no competing financial interests.

## Funding

This work was supported by grants from the Wellcome Trust [WT093811MA to T.B.]; the James S. McDonnell Foundation [to A.M.O. and J.D.P.]; the UK Medical Research Council [U.1055.01.002.00001.01 to A.M.O. and J.D.P.]; the Canada Excellence Research Chairs Program [to A.M.O.]; the National Institute for Health Research Cambridge Biomedical Research Centre [to J.D.P.]; and the National Institute for Health Research Senior Investigator Award [to J.D.P.].

## Author contributions

S.C. designed the study, collected EEG data from healthy volunteers, analysed all the EEG data, and wrote the paper. P.F. and E.K. collected EEG and fMRI data from patients. P.F. and M.M.M. analysed patient fMRI data. J.A. conducted neurological evaluations of patients and jointly oversaw the clinical care of patients with J.D.P., who chaired the research group, and was responsible for research and clinical governance issues. A.M.O. and T.B. provided conceptual input during the design of the study.

## Figures and Tables

**Table 1:** Demographic and assessment details of patients from whom EEG data was analysed.

Patient	Post-ictal interval (months)	Gender	Age at assessment (years)	Aetiology	Diagnosis	CRS-R	Exogenous attention (EEG)	Endogenous attention (EEG)	Command following (fMRI)	Command following (CRS-R)	Cortical atrophy score
P1	4	M	23	TBI	VS	7	Yes	Yes	Yes (SMA)	No	1.0
P2	28	M	31	TBI	VS	7	No	No	No	No	4.0
P3	22	M	24	TBI	VS	8	No	No	No	No	4.0
P4	15	M	38	TBI	VS	8	No	No	Yes (SMA)	No	3.5
P5	18	M	20	TBI	VS	7	No	No	Yes (SMA)	No	4.0
P6	14	F	25	TBI	VS	7	No	No	Yes (PMC)	No	1.0
P7	8	M	36	TBI	VS	7	No	No	No	No	2.0
P8	11	M	23	Anoxia	VS	7	No	No	No	No	3.5
P9	14	M	40	Anoxia	VS	7	No	No	No	No	4.0
P10	68	M	29	TBI	MCS	10	Yes	No	No	Yes	3.0
P11	6	F	29	Anoxia	MCS	9	Yes	No	Yes (IPS)	No	1.5
P12	11	F	20	TBI	MCS	11	No	No	Artefact	No	4.0
P13	6	F	36	Anoxia	MCS	8	No	No	No	No	4.0
P14	35	M	52	TBI	MCS	13	No	No	Yes (SMA)	Yes	2.0
P15	4	M	19	TBI	MCS	12	No	No	No	No	2.5
P16	86	M	37	TBI	MCS	12	No	No	No	No	3.0
P17	13	M	45	TBI	MCS	14	No	No	Yes (SMA)	Yes	1.0
P18	24	F	60	TBI	MCS	16	No	No	No	Yes	1.5
P19	4	M	24	TBI	MCS	13	No	No	Yes (SMA)	Yes	2.0
P20	10	M	41	TBI	MCS	19	Yes	No	Yes (SMA)	Yes	2.0
P21	7	M	38	TBI	MCS	14	No	No	No	Yes	2.0

**Table 2:** CRS-R subscores of patients.

Patient	Auditory	Visual	Motor	Oromotor	Communication	Arousal
P1	1	1	2	1	0	2
P2	1	1	2	1	0	2
P3	1	1	2	2	0	2
P4	1	1	2	2	0	2
P5	1	1	2	1	0	2
P6	1	1	2	1	0	2
P7	1	1	2	1	0	2
P8	1	1	2	1	0	2
P9	1	1	2	1	0	2
P10	3	2	2	1	0	2
P11	1	3	2	1	0	2
P12	1	3	3	2	0	2
P13	1	2	2	1	0	2
P14	3	4	2	2	0	2
P15	2	3	3	2	0	2
P16	2	3	3	2	0	2
P17	3	3	3	2	0	3
P18	3	5	3	1	1	3
P19	3	5	2	1	0	2
P20	4	5	6	1	0	3
P21	3	4	4	1	0	2
