# Supplementary material for: Dissociable endogenous and exogenous attention in disorders of consciousness
Source: Neuroimage Clin. 2013 Oct 16;3:450–61. doi: 10.1016/j.nicl.2013.10.008 (PMC3830059; doi:10.1016/j.nicl.2013.10.008)
Supplement: Inline Supplementary Table S1 — Target and distractor words used in the experimental task. [file mmc1.docx]

**Supplementary Table 1 – Target and distractor words used in the experimental task.** Target words are indicated in italics.

| **Word** | **Duration (ms)** | **Word** | **Duration (ms)** |
| --- | --- | --- | --- |
| *yes* | 525 | rat | 618 |
| *no* | 520 | rice | 637 |
| gown | 571 | robe | 543 |
| mop | 576 | rock | 587 |
| moss | 590 | roof | 591 |
| moth | 499 | rope | 501 |
| mug | 542 | rug | 540 |
| nail | 595 | shed | 554 |
| newt | 637 | ship | 525 |
| oak | 470 | sock | 633 |
| oat | 537 | tape | 520 |
| owl | 599 | toad | 543 |
| ox | 572 | tram | 579 |
| pan | 569 | tub | 455 |
| pear | 503 | van | 596 |
| pea | 480 | vat | 592 |
| pen | 450 | vine | 638 |
| pig | 490 | wasp | 627 |
| pill | 528 | wig | 549 |
| pine | 607 | wine | 535 |
| pin | 478 | wolf | 521 |
| plum | 537 | wood | 443 |
| pram | 552 | wool | 572 |
| punt | 582 | worm | 625 |
| raft | 639 | wren | 507 |
| rake | 600 | yak | 540 |
|  |  | **Mean** | **554** |
