# Supplementary material for: Dissociable endogenous and exogenous attention in disorders of consciousness
Source: Neuroimage Clin. 2013 Oct 16;3:450–61. doi: 10.1016/j.nicl.2013.10.008 (PMC3830059; doi:10.1016/j.nicl.2013.10.008)
Supplement: Inline Supplementary Table S2 [file mmc2.docx]

**Supplementary Table 2 – Results of data preprocessing in healthy volunteers (HV1-8) and selected patients (P1, P10, P11 and P20).**

| **Subject** | **Channels Rejected** | **Trials Rejected** | **ICA Components Rejected** | **Explicit Target Trials** | **Implicit Target Trials** | **Distractor Trials** |
| --- | --- | --- | --- | --- | --- | --- |
| **HV1** | 0 | 12 | 26 | 234 | 233 | 441 |
| **HV2** | 17 | 20 | 22 | 237 | 232 | 633 |
| **HV3** | 9 | 4 | 21 | 241 | 241 | 632 |
| **HV4** | 3 | 0 | 23 | 237 | 237 | 669 |
| **HV5** | 2 | 0 | 20 | 243 | 243 | 660 |
| **HV6** | 2 | 0 | 10 | 291 | 291 | 487 |
| **HV7** | 1 | 6 | 9 | 290 | 291 | 489 |
| **HV8** | 5 | 9 | 7 | 293 | 292 | 542 |
| **P1** | 0 | 9 | 5 | 288 | 288 | 489 |
| **P10** | 11 | 11 | 41 | 294 | 295 | 474 |
| **P11** | 0 | 191 | 28 | 282 | 282 | 479 |
| **P20** | 2 | 58 | 47 | 277 | 280 | 514 |
